# Supplementary material for: Adoptive macrophage directed photodynamic therapy of multidrug-resistant bacterial infection
Source: Nat Commun. 2023 Nov 9;14:7251. doi: 10.1038/s41467-023-43074-9 (PMC10636156; doi:10.1038/s41467-023-43074-9)
Supplement: Supplementary file 1 — Supplementary Information [file 41467_2023_43074_MOESM1_ESM.pdf]

Adoptive Macrophage Directed Photodynamic Therapy of Multidrug-Resistant Bacterial Infection

Zehui Wang [1,3], Anhua Wu [2,3], Wen Cheng [2,3], Yuhe Li [2,3], Dingxuan Li [1], Lai Wang [1], Xinfu Zhang [1,*] and Yi Xiao [1]

[1] State Key Laboratory of Fine Chemicals, Frontiers Science Center for Smart Materials Oriented Chemical Engineering, Dalian University of Technology, Dalian 116024, China.

[2] Department of Neurosurgery, Shengjing Hospital of China Medical University, Shenyang 110055, China.

[3] These authors contributed equally: Zehui Wang, Anhua Wu, Wen Cheng, Yuhe Li.

1. Synthesis of Lyso700D.

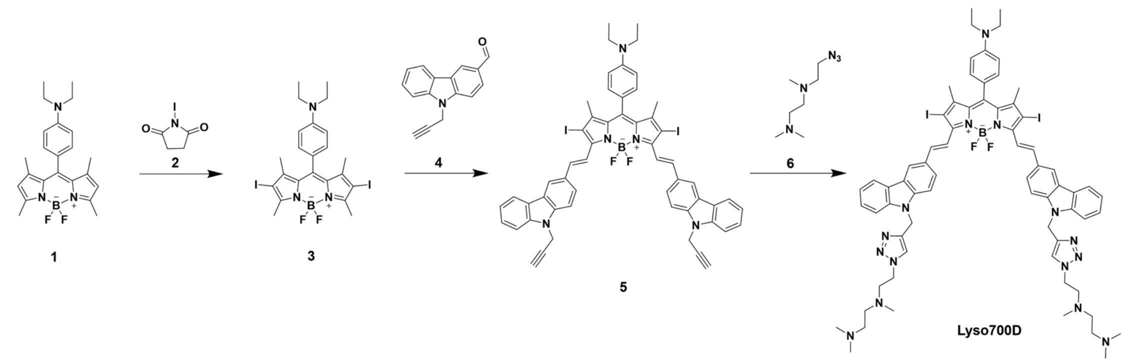

Supplementary Figure S1. Synthetic route of **Lyso700D**.

compound 1, **3**, and **4** were synthesized according to literature methods[1,2].

1.1 Synthesis of **5**.

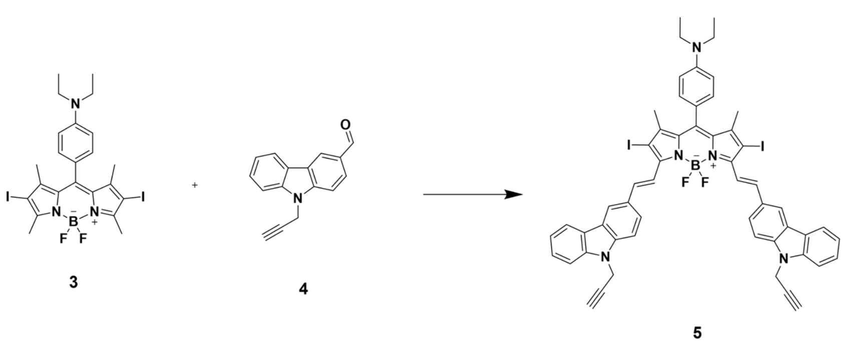

compound 3 (500 mg, 772.68 μmol) and **compound 4** (369 mg, 1.7 mmol) were dissolved in 10 ml of anhydrous toluene, and 0.5 ml of piperidine and 0.5 ml of glacial acetic acid were added successively. Reflux at boiling point. The progress of the reaction was monitored by TLC. The solution was diluted with deionized water and extracted with CH_2Cl_2 (3X). The combined organic solvent layer was dried with $MgSO_4$ and concentrated. The crude product was purified by flash chromatography (silica gel) to afford a green solid **compound 5** (0.587 g, 554.73 μmol) in a 70.50% yield. ^{1}H NMR (400 MHz, DMSO) δ 8.47 (s, 2H), 8.39 – 8.28 (m, 4H), 7.89 – 7.78 (m, 4H), 7.70 (dd, J = 24.9, 12.4 Hz, 4H), 7.54 (t, J = 7.7 Hz, 2H), 7.28 (d, J = 7.5 Hz, 2H), 7.15 (s, 2H), 6.86 (d, J = 8.4 Hz, 2H), 5.36 (d, J = 1.7 Hz, 4H), 3.43 (d, J = 6.8 Hz, 4H), 3.29 (s, 2H), 1.61 (s, 6H), 1.16 (s, 6H). HRMS (ESI, m/z): $[M+H]^+$ calcd for $C_{55}H_{45}BF_2I_2N_5$, 1078.1820; found, 1078.1810. $[M+Na]^+$ calcd for $C_{55}H_{44}BF_2I_2N_5Na$, 1100.1639; found, 1100.1624. $[M+K]^+$ calcd for $C_{55}H_{44}BF_2I_2N_5K$, 1116.1379; found, 1116.1367.

1.2 Synthesis of **Lso700D**.

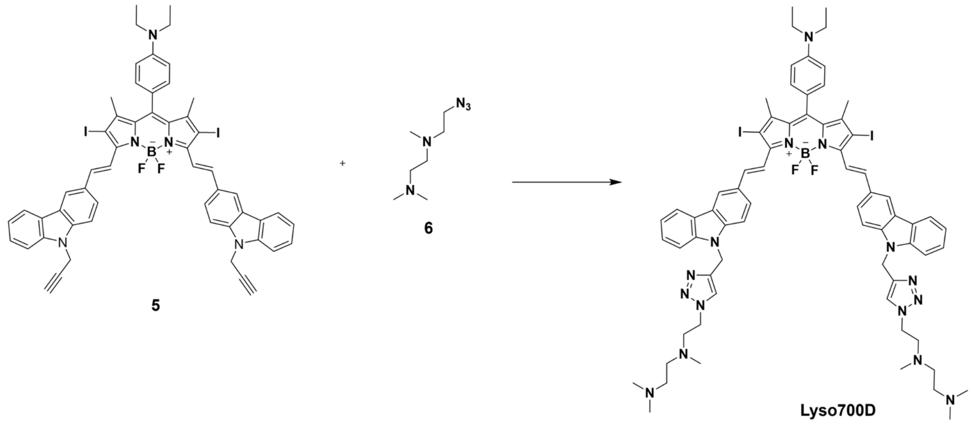

compound 5 (25 mg, 23.20 μmol), **compound 6** (4.77 mg, 2.84 μmol), sodium ascorbate (0.41 mg, 2.32 μmol), $CuSO_4$ $5H_2O$ (0.053 mg, 2.32 μmol) It was added to a mixture of THF and H_2O (v/v 3 : 1), under argon protection, stirred at room temperature for 24 h, the solvent was distilled off under reduced pressure, and purified by silica gel column chromatography, developing solvent CH_2Cl_2: EtOH (35 : 1, v/v) to obtain the dark green solid **Lyso700D** (22 mg, 16.20 μmol) in a 69.81% yield. ^{1}H NMR (400 MHz, $CDCl_3$) δ 8.25 (d, J = 14.9 Hz, 4H), 8.16 – 8.05 (m, 4H), 7.91 – 7.74 (m, 6H), 7.65 (d, J = 16.6 Hz, 2H), 7.53 (t, J = 7.5 Hz, 2H), 7.23 (d, J = 7.4 Hz, 2H), 7.05 (d, J = 8.4 Hz, 2H), 6.79 (d, J = 7.9 Hz, 2H), 5.62 (s, 4H), 4.38 (s, 4H), 3.45 (d, J = 7.1 Hz, 4H), 2.71 (s, 4H), 2.54 (s,

8H), 2.11 (s, 12H), 2.01 (s, 6H), 1.31 (s, 6H), 1.28 – 1.19 (m, 6H). HRMS (ESI, m/z): $[M+H]^+$ calcd for $C_{69}H_{79}BF_2I_2N_{15}$, 1420.4788; found, 1420.4747. $[M+2H]^{2+}$ calcd for $C_{69}H_{80}BF_2I_2N_{15}$, 710.7430; found, 710.7410.

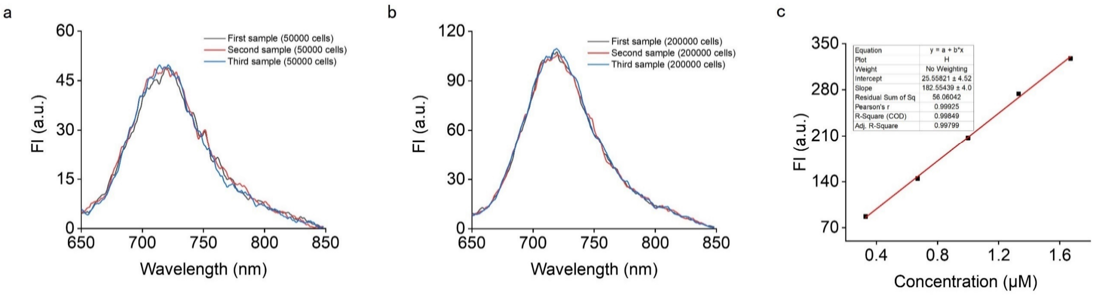

Supplementary Figure S2. (a) Fluorescence intensity of **Lyso700D** in RAW264.7 (5×10^4 cells). (b) Fluorescence intensity of **Lyso700D** in RAW264.7 (2×10^5 cells). (c) Standard curve of fluorescence intensity fitting of **Lyso700D** in ethanol at different concentrations.

Supplementary Table S1. Calculation of the average number of molecules in each RAW264.7 cell.

Sample	*in vitro*			*in vivo*		
FI	48.96	48.58	49.18	107.58	105.02	109.76
c	0.13	0.13	0.13	0.45	0.44	0.46
$n_{single\ cell}$	2.56×10^{-15}	2.52×10^{-15}	2.59×10^{-15}	2.25×10^{-15}	2.18×10^{-15}	2.31×10^{-15}
$n_{single\ cell}$		2.56×10^{-15}			2.24×10^{-15}	
std		3.34×10^{-17}			6.51×10^{-17}	

Note: FI is the fluorescence intensity of each sample in ethanol, *a.u.*; c is the concentration of **Lyso700D** in ethanol, μM; $n_{single\ cell}$ is mole of Lyso700D in each RAW264.7 cell, mol; $n_{single\ cell}$ is average mole of **Lyso700D** in each RAW264.7 cell, mol; std is the standard deviation of the molar amount of **Lyso700D** in each RAW264.7 cell was calculated.

Supplementary Table S2. A summary of the reported doses and the corresponding references for use in epidermal wound models.

Model	Epidermal wound	Epidermal wound	Epidermal wound	Epidermal wound	Epidermal wound	Epidermal wound	Epidermal wound
Equivalent dose	3.66 mg/kg	1 mg/kg	4 mg/kg	24 mg/kg	2 mg/kg	1 mg/kg	8.53 μg/kg
ref	37	38	41	42	43	44	45

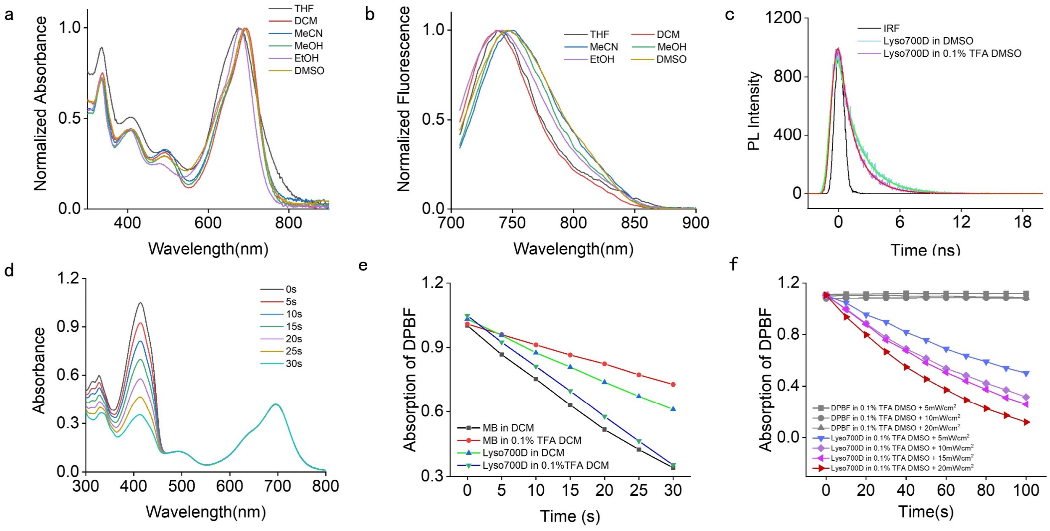

Supplementary Figure S3. (a) The absorption spectrum of **Lyso700D** in different organic solvents. (b) The emission spectrum of **Lyso700D** in different organic solvents. (c) Fluorescence decay trace of **Lyso700D** in DCM at 736 nm. (d) $^1O_2$ generation of **Lyso700D** trapped with DPBF in DCM (0.01% TFA) irradiated with 660 nm (1 mW/cm^2, 5×10^{-3} J/cm^2). (e) $^1O_2$ generation of **Lyso700D** and MB trapped with DPBF in DCM (0.01% TFA) irradiated with 660 nm (1 mW/cm^2, 5×10^{-3} J/cm^2). (f) $^1O_2$ generation of **Lyso700D** under 660 nm LED light over different laser power densities.

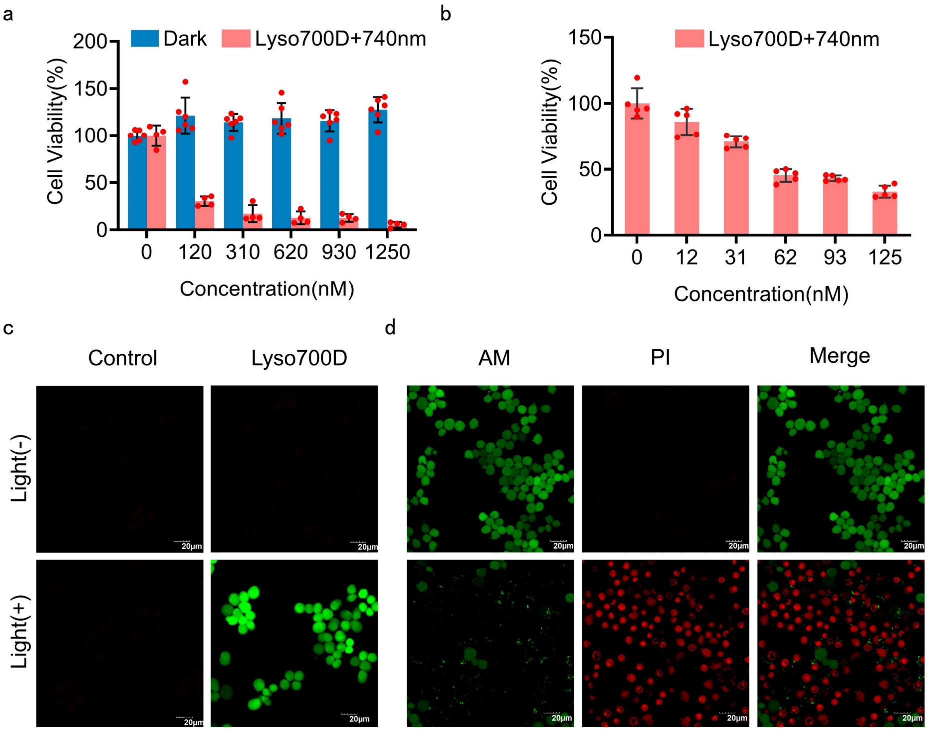

Supplementary Figure S4. (a) Cell viability of D-RAWs with or without irradiation at 740 nm (30 mW/cm^2, 27 J/cm^2). (b) Concentration-dependent phototoxicity of **Lyso700D** under 740 nm (30 mW/cm^2, 27 J/cm^2). (c) Detection of D-RAWs intracellular ROS generated by 740 nm (30 mW/cm^2, 27 J/cm^2). (d) Confocal images of D-RAWs containing Calcein-AM-PI with or without 740 nm light irradiation (Calcein-AM was collected at 500 nm-550 nm by excitation at 488 nm. PI was collected at 565 nm-650 nm by excitation at 559 nm.). Bar: 20 μm.

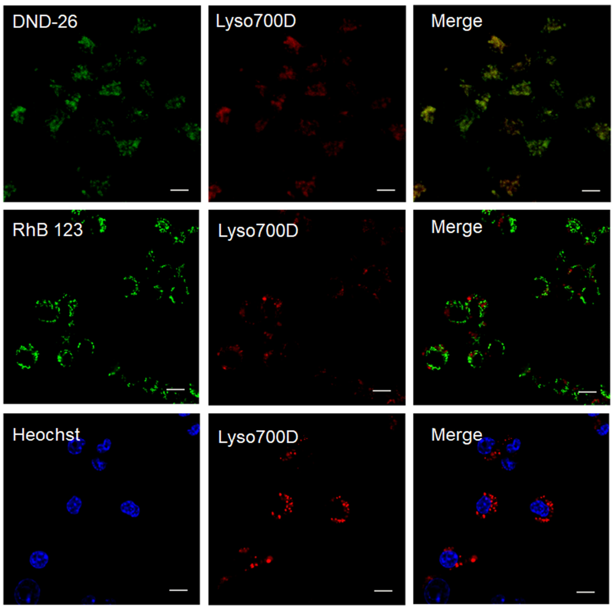

Supplementary Figure S5. Confocal imaging of RAWs costained with **Lyso700D** and DND-26, Rhodamine 123, or Heochst respectively. Bar: 10 μm.

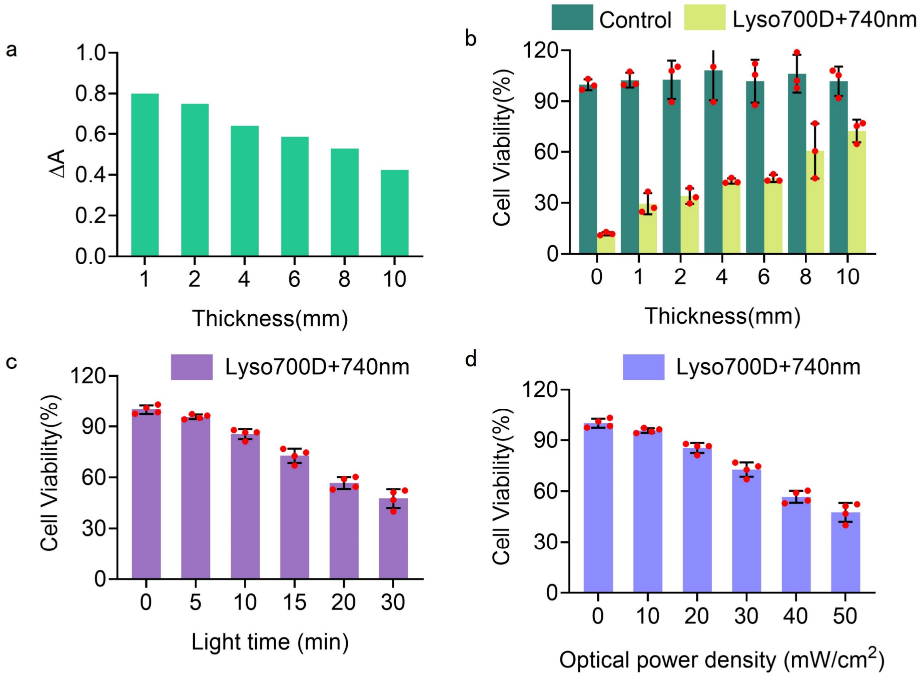

Supplementary Figure S6. (a) The absorption decay of DPBF induced by **Lyso700D** (740nm, 30 mW/cm^2, 0.3 J/cm^2) over chicken breast tissues with different thicknesses. (b) Cell viability of **Lyso700D** covered with different thicknesses of chicken breast tissue under 740 nm (30 mW/cm^2, 27 J/cm^2). (c) Viability of D-RAWs upon irradiation with 740 nm LED over different times. (d) Viability of D-RAWs after irradiation with 740 nm LED under different laser power.

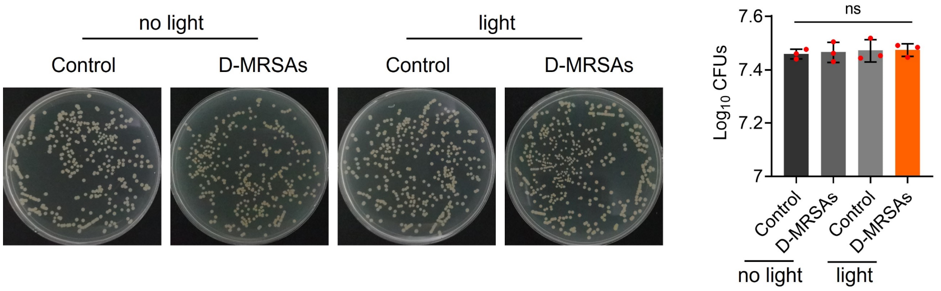

Supplementary Figure S7. Survival of MRSA post phototherapy using freshly prepared D-MRSAs. Statistical analysis was performed by a two-tailed unpaired t test. ns: no significant difference; * $P < 0.05$; ** $P < 0.01$; *** $P < 0.001$.

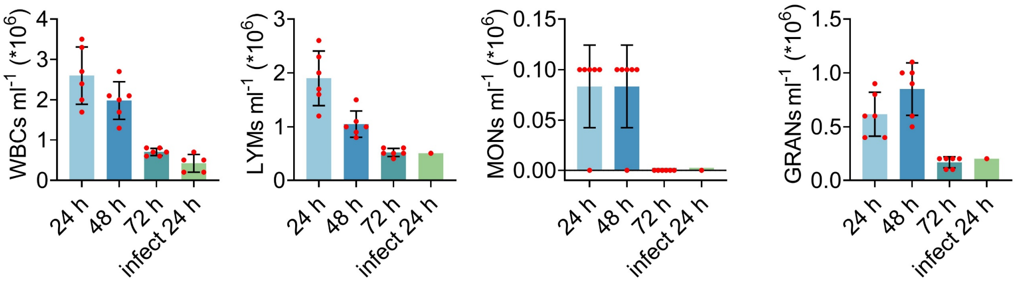

Supplementary Figure S8. Levels of WBCs, LYMs, MONs, and GRANs of mice in the blood after intraperitoneal injection of CY for 3 consecutive days.

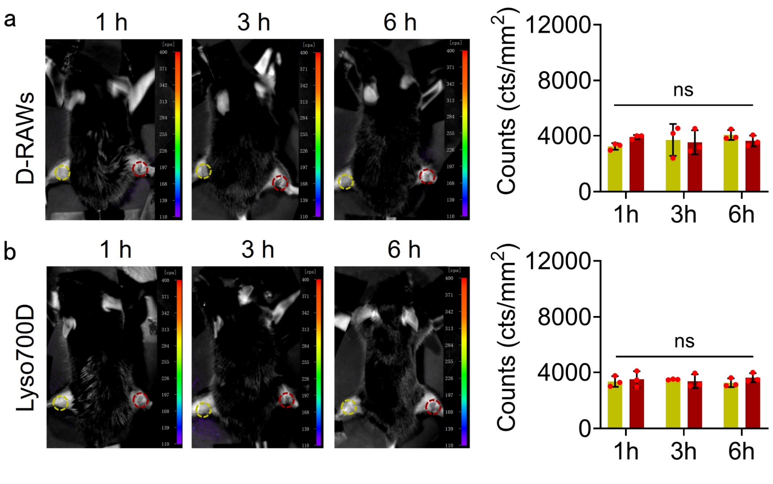

Supplementary Figure S9. (a) Fluorescence images of infected mice at 1, 3 and 6 h post injecting D-RAWs and statistical analysis of the fluorescence intensity of the wounds on legs. (b) Fluorescence images of infected mice at 1, 3 and 6 h post injecting **Lyso700D** and statistical analysis of the fluorescence intensity of the wounds on legs. Statistical analysis was performed by a two-

tailed unpaired t test. ns: no significant difference; * $P < 0.05$; ** $P < 0.01$; *** $P < 0.001$.

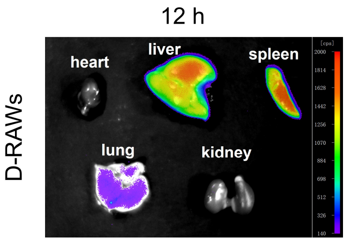

Supplementary Figure S10. Ex-vivo fluorescence imaging of organs from healthy mice 12 h after the administration of D-RAWs.

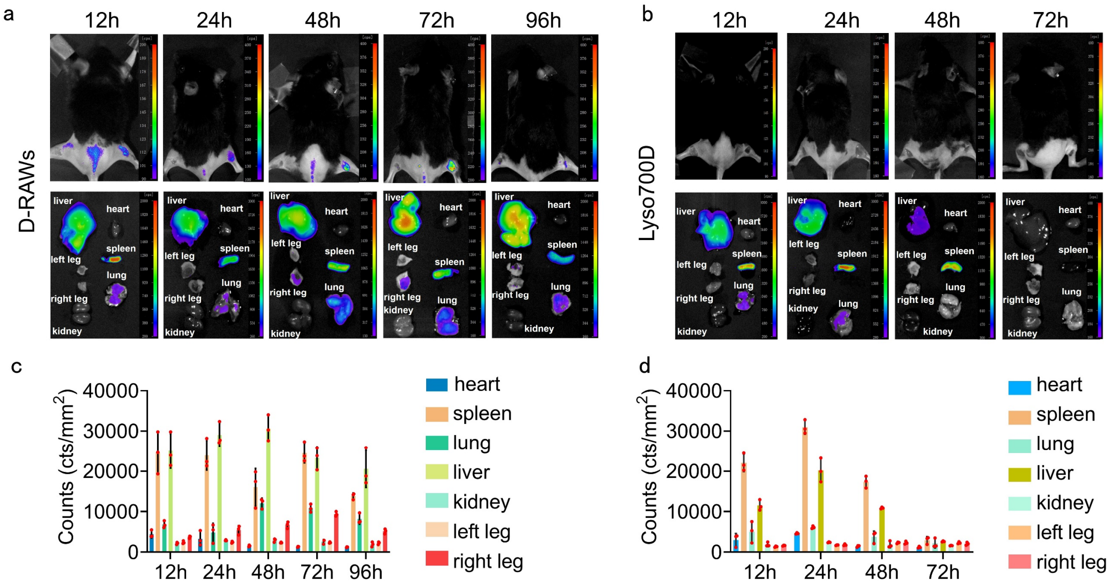

Supplementary Figure S11 (a, b) D-RAWs (a) and **Lyso700D** (b) real-time fluorescence intensity distribution of major organs and legs in vivo. (c, d) D-RAWs (c) and **Lyso700D** (d) real-time fluorescence intensity statistics of major organs and legs in vivo in an immunodeficient mice epidermal inflammation model.

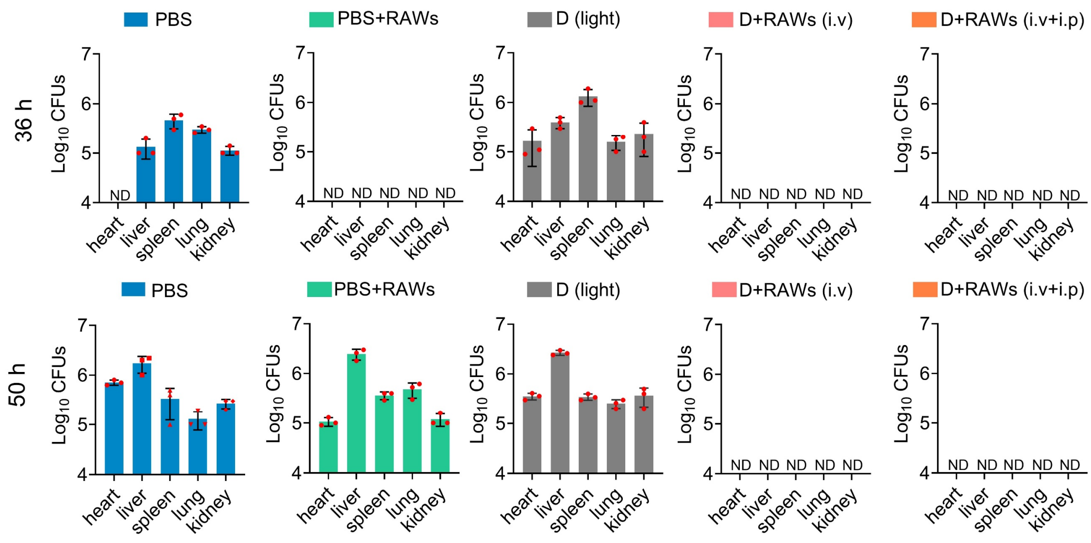

Supplementary Figure S12 Bacterial burden statistics of major organs at 36 h and 50 h post phototherapy in an immunodeficient mice epidermal inflammation model.

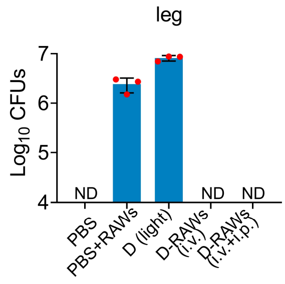

Supplementary Figure S13 Bacterial burden statistics of survived mice right leg each group at 30 days in an immunodeficient mice epidermal inflammation model.

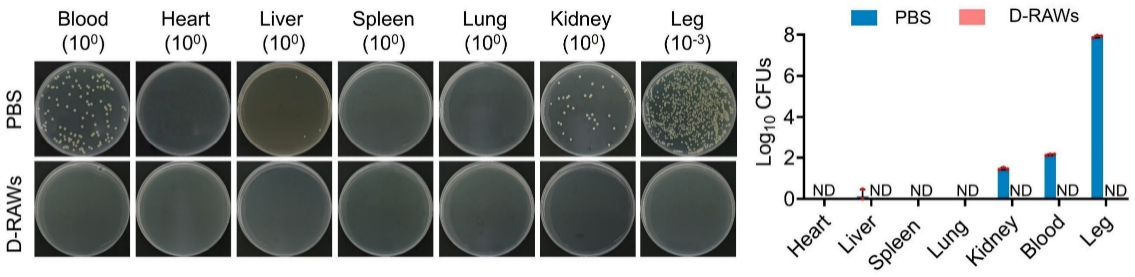

Supplementary Figure S14 Bacterial burden of major organs, blood, and right leg at 50 h post phototherapy in an immunocompetent mice epidermal inflammation model.

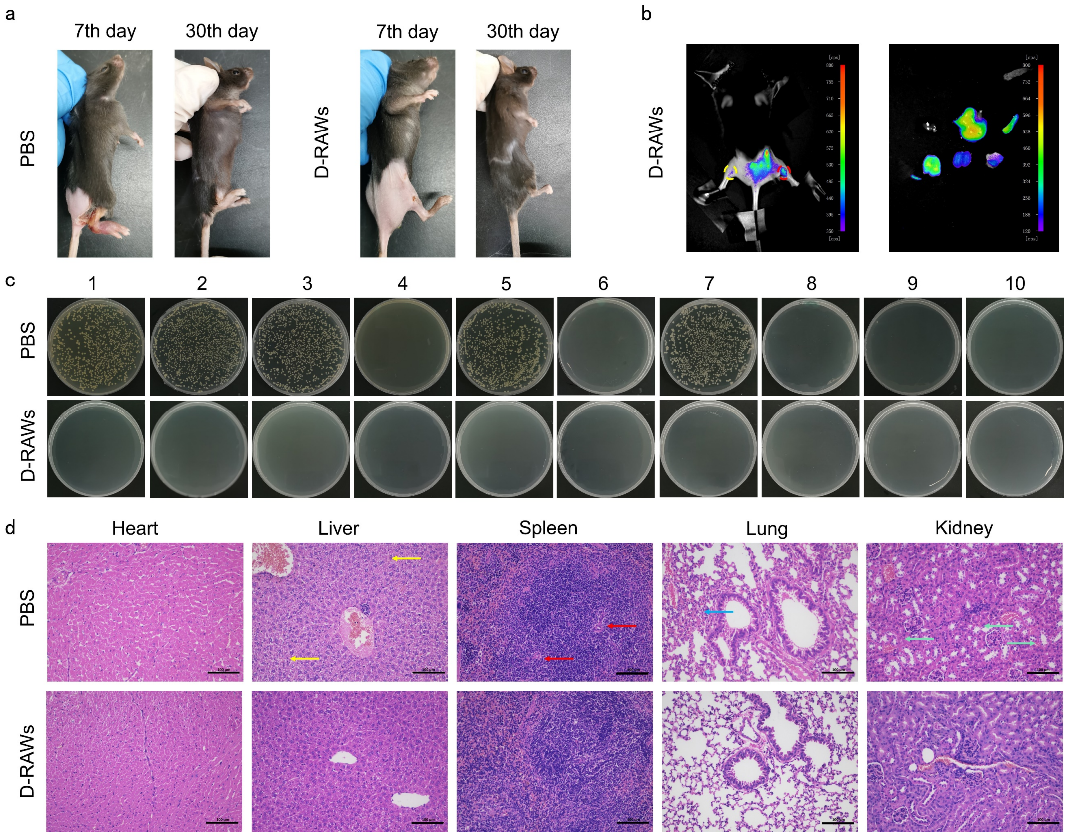

Supplementary Figure S15 (a) The real pictures of infected wounds at different times in each group of mice in an immunocompetent mice epidermal inflammation model. (b) Fluorescence images of infected mice post 12 hours injecting D-RAWs. (c) Bacterial burden in the leg of each surviving mouse by PBS and D-RAWs after 30 days in an immunocompetent mice epidermal inflammation model. (d) H&E staining of major organs in each group after 30 days in an immunocompetent mice epidermal inflammation model. Bar: 100 nm.

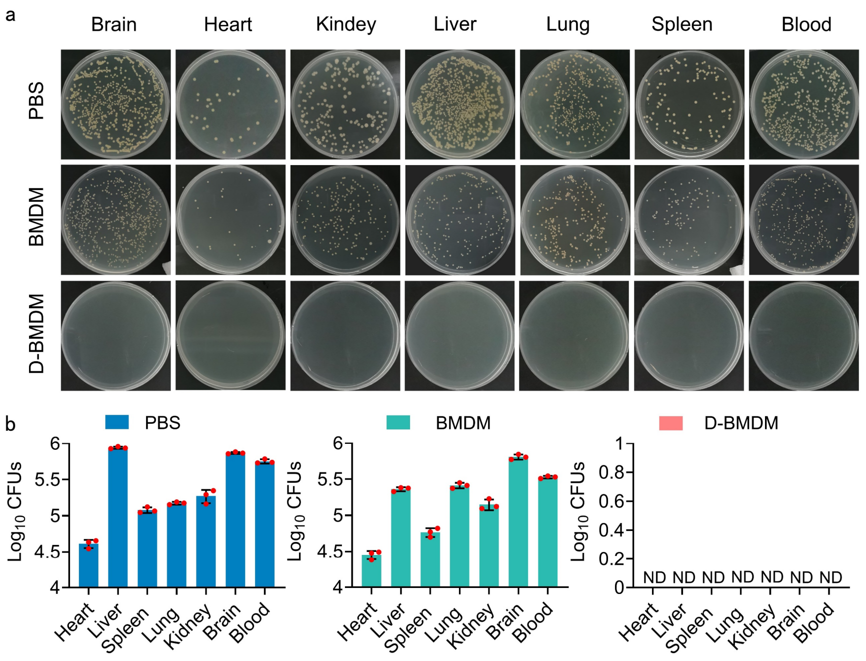

Supplementary Figure S16 Bacterial burden of major organs, blood, and brain at 26 h post phototherapy in an immunodeficient rats' meningitis model.

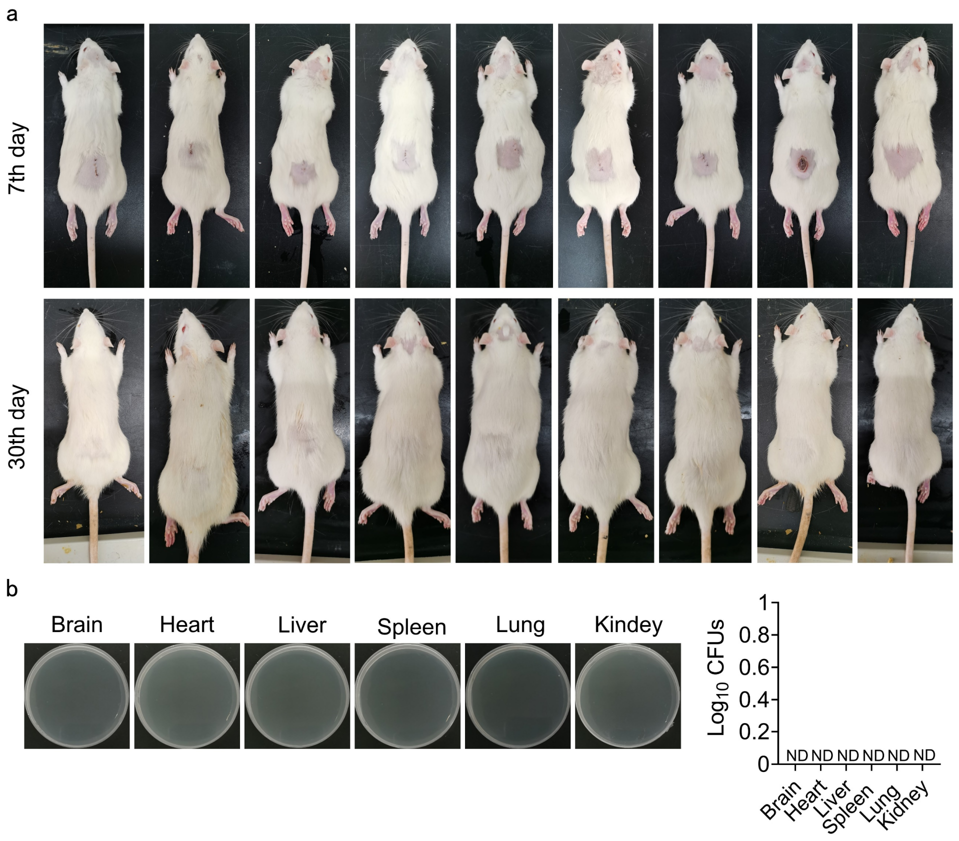

Supplementary Figure S17 (a) The real pictures of infected wounds at different times in each group

of rats in an immunodeficient rats' meningitis model. (b) Bacterial burden of brain and major organs D-BMDMs group rats at 30 days in an immunodeficient rats' meningitis model.

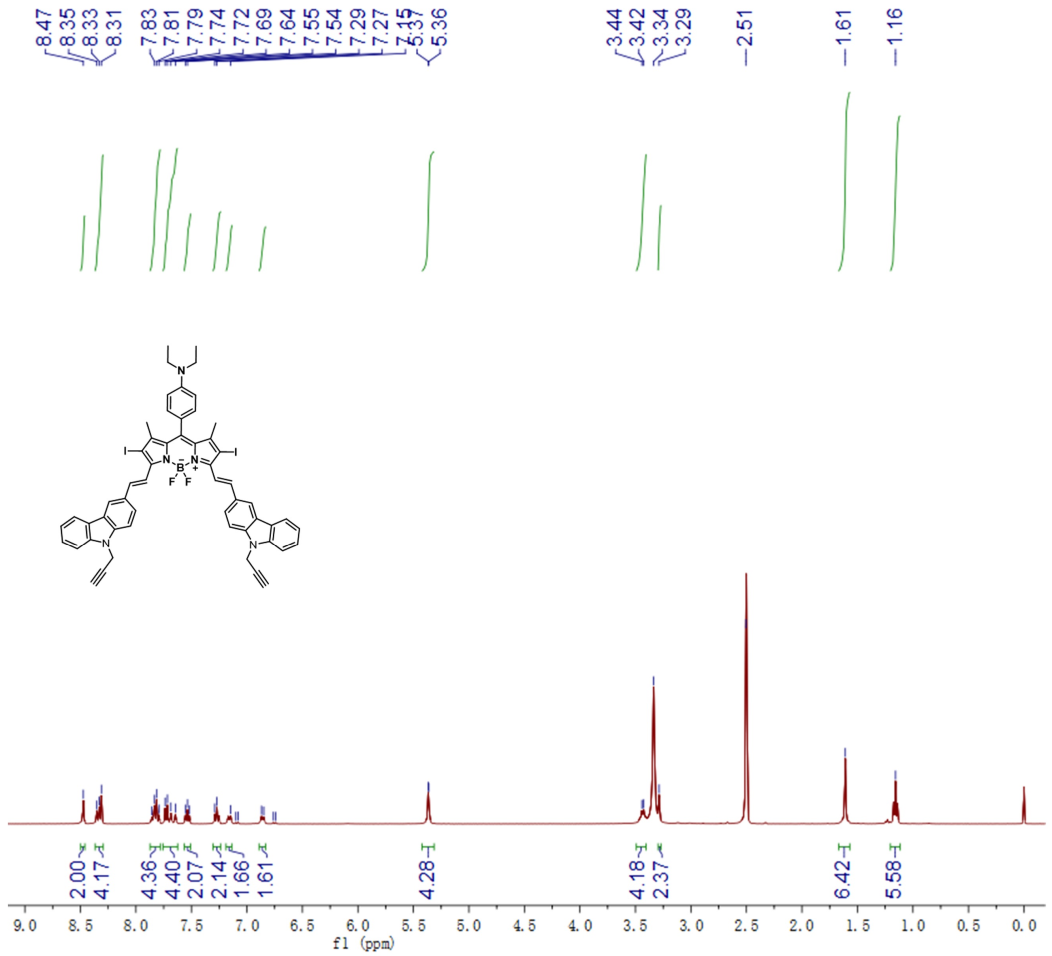

Supplementary Figure S18 ^{1}H-NMR spectrum of **compound 5** DMSO.

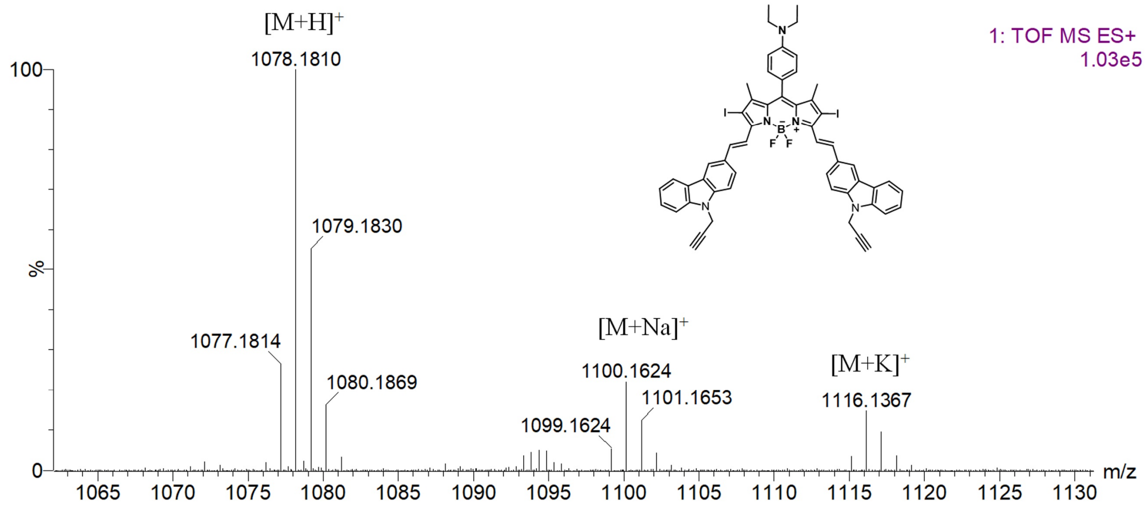

Supplementary Figure S19 HRMS spectrum of **compound 5**.

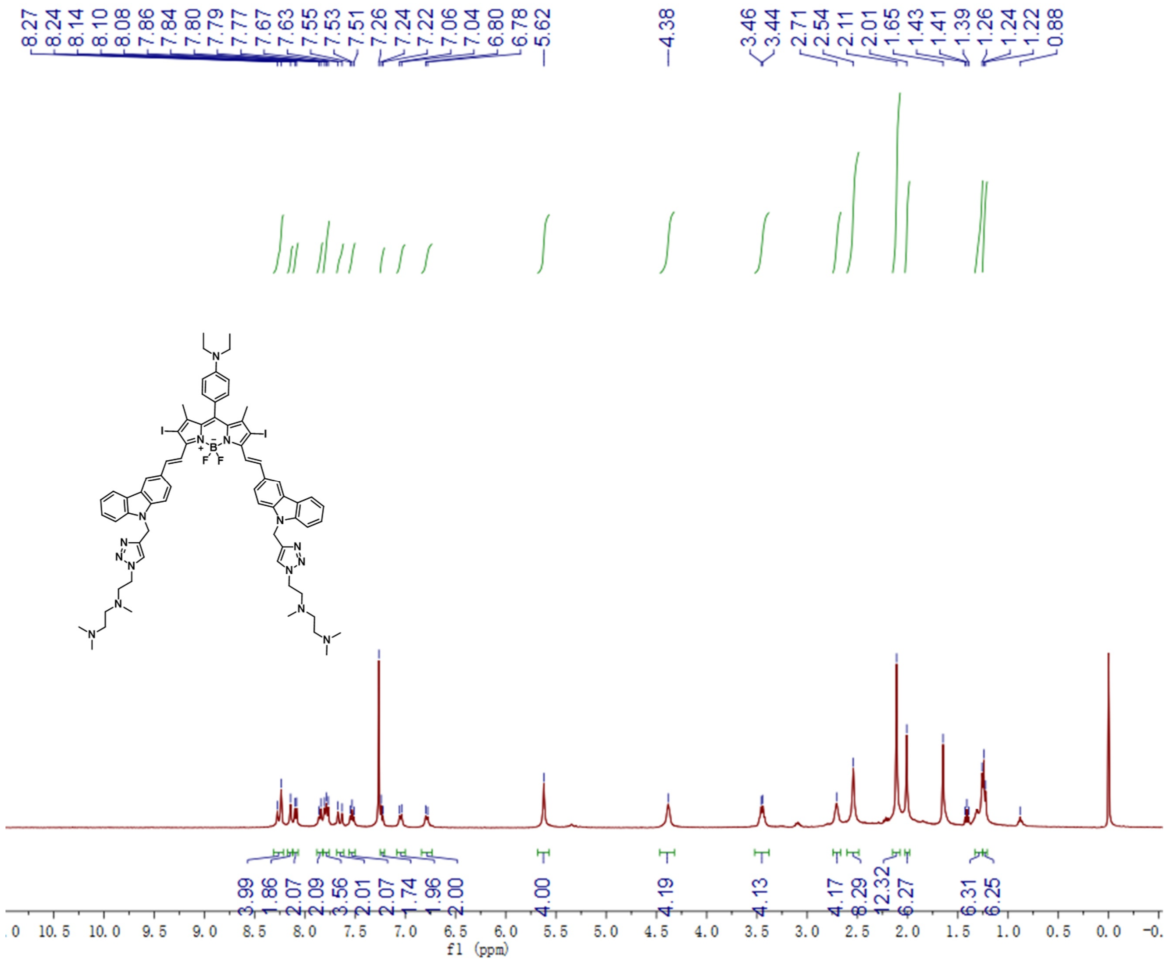

Supplementary Figure S20 ^{1}H-NMR spectrum of **Lyso700D** $CDCl_3$.

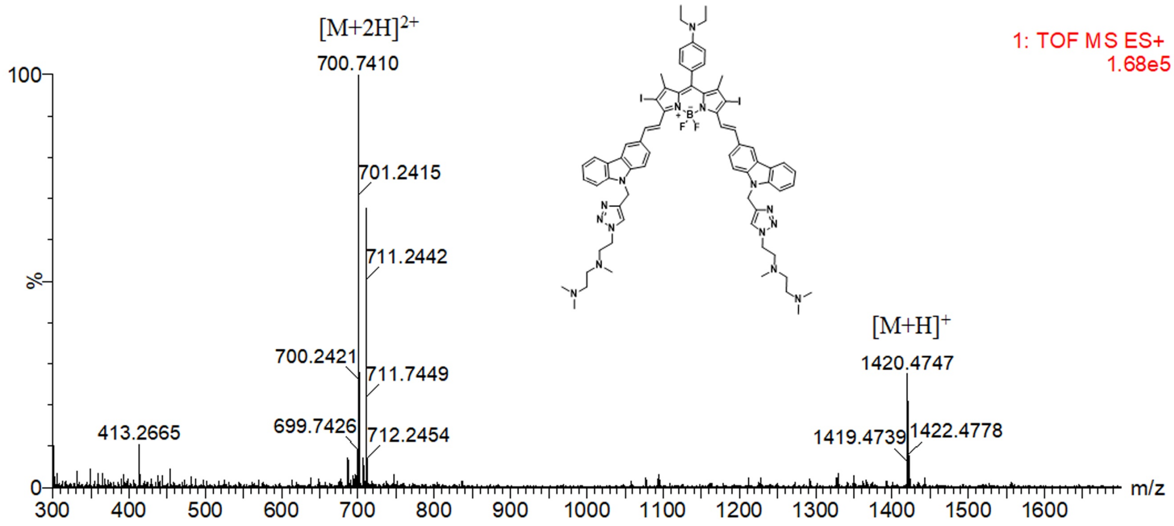

Supplementary Figure S21 HRMS spectrum of **Lyso700D**.

References:

1 Xiong, H., Zhou, K., Yan, Y., Miller, J.B. & Siegwart, D.J. Tumor-Activated Water-Soluble Photosensitizers for Near-Infrared Photodynamic Cancer Therapy. *ACS Appl Mater Interfaces* 10, 16335-16343 (2018).

2. Zhang, X., Wang, C., Jin, L., Han, Z. & Xiao, Y. Photostable bipolar fluorescent probe for video tracking plasma membranes related cellular processes. *ACS Appl Mater Interfaces* 6, 12372-12379 (2014).

3. Frimodt-Møller, N., Knudsen, J. & Espersen, F. *in Handbook of Animal Models of Infection* 127–136 (1999).

4. McVicker, G. et al. Clonal expansion during Staphylococcus aureus infection dynamics reveals the effect of the antibiotic intervention. *PLoS Pathog.* 10, e1003959 (2014).

5. Zhang, L. et al. High-throughput synergy screening identifies microbial metabolites as combination agents for the treatment of fungal infections. *Proc. Natl Acad. Sci. USA* 104, 4606–4611 (2007).
