# Supplementary material for: Adoptive macrophage directed photodynamic therapy of multidrug-resistant bacterial infection
Source: Nat Commun. 2023 Nov 9;14:7251. doi: 10.1038/s41467-023-43074-9 (PMC10636156; doi:10.1038/s41467-023-43074-9)
Supplement: Supplementary file 3 — Reporting Summary [file 41467_2023_43074_MOESM3_ESM.pdf]

nature portfolio

Corresponding author(s): Xinfu Zhang

Last updated by author(s): Oct 10, 2023

Reporting Summary

Nature Portfolio wishes to improve the reproducibility of the work that we publish. This form provides structure for consistency and transparency in reporting. For further information on Nature Portfolio policies, see our Editorial Policies and the Editorial Policy Checklist.

Statistics

For all statistical analyses, confirm that the following items are present in the figure legend, table legend, main text, or Methods section.

n/a	Confirmed	
☐	☒	The exact sample size (*n*) for each experimental group/condition, given as a discrete number and unit of measurement
☐	☒	A statement on whether measurements were taken from distinct samples or whether the same sample was measured repeatedly
☐	☒	The statistical test(s) used AND whether they are one- or two-sided<br>*Only common tests should be described solely by name; describe more complex techniques in the Methods section.*
☐	☒	A description of all covariates tested
☐	☒	A description of any assumptions or corrections, such as tests of normality and adjustment for multiple comparisons
☐	☒	A full description of the statistical parameters including central tendency (e.g. means) or other basic estimates (e.g. regression coefficient) AND variation (e.g. standard deviation) or associated estimates of uncertainty (e.g. confidence intervals)
☐	☒	For null hypothesis testing, the test statistic (e.g. *F*, *t*, *r*) with confidence intervals, effect sizes, degrees of freedom and *P* value noted<br>*Give P values as exact values whenever suitable.*
☒	☐	For Bayesian analysis, information on the choice of priors and Markov chain Monte Carlo settings
☒	☐	For hierarchical and complex designs, identification of the appropriate level for tests and full reporting of outcomes
☐	☒	Estimates of effect sizes (e.g. Cohen's *d*, Pearson's *r*), indicating how they were calculated

Our web collection on statistics for biologists contains articles on many of the points above.

Software and code

Policy information about availability of computer code

Data collection	UV-vis-NIR spectra were performed on Agilent 8453 UV-visible or Lambda 750S spectroscopy;<br>Fluorescence images were obtained with a confocal laser scanning microscope (Olympus Fluoview FV1000);<br>Small animals' fluorescence imaging was carried out by NightOWL II LB983 living imaging system.
Data analysis	The statistical analysis was performed with GraphPad Prism and Origin 2018 software (OriginLab, Northhampton, MA);<br>The fluorescence images statistical analysis was performed with Olympus FV1000 software;<br>Student's t test was used to evaluate the statistical significance.

For manuscripts utilizing custom algorithms or software that are central to the research but not yet described in published literature, software must be made available to editors and reviewers. We strongly encourage code deposition in a community repository (e.g. GitHub). See the Nature Portfolio guidelines for submitting code & software for further information.

Data

Policy information about availability of data

All manuscripts must include a data availability statement. This statement should provide the following information, where applicable:

- Accession codes, unique identifiers, or web links for publicly available datasets
- A description of any restrictions on data availability
- For clinical datasets or third party data, please ensure that the statement adheres to our policy

All data generated or analysed during this study are included in this published article (and its supplementary information files). Source data are provided with this paper.

Research involving human participants, their data, or biological material

Policy information about studies with human participants or human data. See also policy information about sex, gender (identity/presentation), and sexual orientation and race, ethnicity and racism.

Reporting on sex and gender	No human study participants are involved in this manuscript.
Reporting on race, ethnicity, or other socially relevant groupings	No human study participants are involved in this manuscript.
Population characteristics	No human study participants are involved in this manuscript.
Recruitment	No human study participants are involved in this manuscript.
Ethics oversight	No human study participants are involved in this manuscript.

Note that full information on the approval of the study protocol must also be provided in the manuscript.

Field-specific reporting

Please select the one below that is the best fit for your research. If you are not sure, read the appropriate sections before making your selection.

☒ Life sciences ☐ Behavioural & social sciences ☐ Ecological, evolutionary & environmental sciences

For a reference copy of the document with all sections, see nature.com/documents/nr-reporting-summary-flat.pdf

Life sciences study design

All studies must disclose on these points even when the disclosure is negative.

Sample size	For all in vitro (cell, bacteria) test, at least 3 samples are used to give average value with significant difference analysis; In flow cytometry experiments,3 samples are used to give average value with significant difference analysis; for in vivo infection model, in infection of epidermal bacterial, 3 samples are used to give average value with significant difference analysis in vivo tracking and bacterial loading test; at least 5 samples are used to give average value with significant difference analysis in the WBCs and LYMs statistical experiment; 10 samples were averaged from the records of survival and body weight of mice for significant difference analysis. In infection of meningitis bacterial, at least 3 samples are used to give average value with significant difference analysis in bacterial loading test; at least 4 samples are used to give average value with significant difference analysis in the WBCs and LYMs statistical experiment; 9 samples were averaged from the records of survival and body weight of mice for significant difference analysis.
Data exclusions	No data exclusions was performed from the analyses in the manuscript.
Replication	All in vitro result are reproducibility in the manuscript. For in vivo assay, 3-10 samples are used for different assays to give solid result with significant difference between control groups and test groups.
Randomization	All data were allocated randomly.
Blinding	Not applicable. All data were allocated randomly by the investigators.

Reporting for specific materials, systems and methods

We require information from authors about some types of materials, experimental systems and methods used in many studies. Here, indicate whether each material, system or method listed is relevant to your study. If you are not sure if a list item applies to your research, read the appropriate section before selecting a response.

Materials & experimental systems

n/a	Involved in the study
☒	☐ Antibodies
☐	☒ Eukaryotic cell lines
☒	☐ Palaeontology and archaeology
☐	☒ Animals and other organisms
☒	☐ Clinical data
☒	☐ Dual use research of concern
☒	☐ Plants

Methods

n/a	Involved in the study
☒	☐ ChIP-seq
☐	☒ Flow cytometry
☒	☐ MRI-based neuroimaging

Eukaryotic cell lines

Policy information about cell lines and Sex and Gender in Research

Cell line source(s)	RAW264.7; Cos-7
Authentication	Short Tandem Repeat identification is correct according to report by supplier.
Mycoplasma contamination	No mycoplasma contamination was detected according to report by supplier.
Commonly misidentified lines (See ICLAC register)	None

Animals and other research organisms

Policy information about studies involving animals; ARRIVE guidelines recommended for reporting animal research, and Sex and Gender in Research

Laboratory animals	C57BL/6 mice,6-7 weeks old; SD rats, 7-8 weeks old
Wild animals	No wild animals were used.
Reporting on sex	Sex was not considered in study design. All mice used in the study are female.
Field-collected samples	None
Ethics oversight	Our research complies with all relevant ethical regulations. All animal studies were performed in accordance with ARRIVE guidelines. All animal experiments were approved by the animal research ethics committee of Dalian University of Technology. The animal study complied with relevant ethical regulations for animal testing and research.

Note that full information on the approval of the study protocol must also be provided in the manuscript.

Plants

Seed stocks	No plant study are involved in this manuscript.
Novel plant genotypes	No plant study are involved in this manuscript.
Authentication	No plant study are involved in this manuscript.

Flow Cytometry

Plots

Confirm that:

☒ The axis labels state the marker and fluorochrome used (e.g. CD4-FITC).

☒ The axis scales are clearly visible. Include numbers along axes only for bottom left plot of group (a 'group' is an analysis of identical markers).

☒ All plots are contour plots with outliers or pseudocolor plots.

☒ A numerical value for number of cells or percentage (with statistics) is provided.

Methodology

Sample preparation	For the retention of Lyso700D in lysosomes over cell proliferation test, Raw264.7 cells in the exponential phase of growth were grown on 6-well plate at 50000 cells per well and culture dishes for 24 h. Then incubated with 250 nM Lyso700D for 3 h at 37 °C under 5% CO2. The cells were washed three times with PBS.
Instrument	LSRII, BD
Software	D-RAWs with different proliferation time was selected for box selection. The fluorescence intensity in the number of cells selected in the frame was calculated by the built-in processing software of the instrument. Finally, the fluorescence intensity of individual cells was calculated as statistical data.
Cell population abundance	At least 5000 cells were selected for fluorescence intensity statistics.
Gating strategy	RAW264.7 cells were selected as negative control. The D-RAWs between different increment were used as positive experimental group.

☐ Tick this box to confirm that a figure exemplifying the gating strategy is provided in the Supplementary Information.
